# Combing Immunoinformatics with Pangenome Analysis To Design a Multiepitope Subunit Vaccine against Klebsiella pneumoniae K1, K2, K47, and K64

**DOI:** 10.1128/spectrum.01148-22

**Published:** 2022-07-12

**Authors:** Zhuohao Wang, Genglin Guo, Quan Li, Pei Li, Min Li, Lu Zhou, Zhongming Tan, Wei Zhang

**Affiliations:** a College of Veterinary Medicine, Nanjing Agricultural University, Nanjing, China; b Key Lab of Animal Bacteriology, Ministry of Agriculture, Nanjing, China; c OIE Reference Lab for Swine Streptococcosis, Nanjing, China; d The Sanya Institute of Nanjing Agriculture University, Sanya, China; e College of Veterinary Medicine, Yangzhou University, Yangzhou, China; f NHC Key Laboratory of Enteric Pathogenic Microbiology, Jiangsu Provincial Center for Disease Control and Prevention, Nanjing, China; University Paris-Saclay, AP-HP Hôpital Antoine Béclère, Service de Microbiologie, Institute for Integrative Biology of the Cell (I2BC), CEA, CNRS

**Keywords:** *Klebsiella pneumoniae*, hypervirulent, carbapenem resistant, pangenome analysis, immunoinformatics, multiepitope subunit vaccine

## Abstract

Klebsiella pneumoniae is an opportunistic Gram-negative bacterium that has become a leading causative agent of nosocomial infections, mainly infecting patients with immunosuppressive diseases. Capsular (K) serotypes K1, K2, K47, and K64 are commonly associated with higher virulence (hypervirulent Klebsiella pneumoniae), and more threateningly, isolates belonging to the last two K serotypes are also frequently associated with resistance to carbapenem (hypervirulent carbapenem-resistant Klebsiella pneumoniae). The prevalence of these isolates has posed significant threats to human health, and there are no appropriate therapies available against them. Therefore, in this study, a method combining immunoinformatics and pangenome analysis was applied for contriving a multiepitope subunit vaccine against these four threatening serotypes. To obtain cross-protection, 12 predicted conserved antigens were screened from the core genome of 274 complete Klebsiella pneumoniae genomes (KL1, KL2, KL47, and KL64), from which the epitopes of T and B cells were extracted for vaccine construction. In addition, the immunological properties, the interaction with Toll-like receptors, and the stability in a simulative humoral environment were evaluated by immunoinformatics methods, molecular docking, and molecular dynamics simulation. All of these evaluations indicated the potency of this constructed vaccine to be an effective therapeutic agent. Lastly, the cDNA of the designed vaccine was optimized and ligated to pET-28a(+) for expression vector construction. Overall, our research provides a newly cross-protective control strategy against these troublesome pathogens and paves the way for the development of a safe and effective vaccine.

**IMPORTANCE** Klebsiella pneumoniae is an opportunistic Gram-negative bacterium that has become a leading causative agent of nosocomial infections. Among the numerous capsular serotypes, K1, K2, K47, and K64 are commonly associated with higher virulence (hypervirulent K. pneumoniae). More threateningly, the last two serotypes are frequently associated with resistance to carbapenem (hypervirulent carbapenem-resistant K. pneumoniae). However, there is currently no therapeutic agent or vaccine specifically against these isolates. Therefore, development of a vaccine against these pathogens is very essential. In this study, for the first time, a method combining pangenome analysis, reverse vaccinology, and immunoinformatics was applied for contriving a multiepitope subunit vaccine against K. pneumoniae isolates of K1, K2, K47, and K64. Also, the immunological properties of the constructed vaccine were evaluated and its high potency was revealed. Overall, our research will pave the way for the vaccine development against these four threatening capsular serotypes of K. pneumoniae.

## INTRODUCTION

Klebsiella pneumoniae is a member of the family *Enterobacteriaceae*, a Gram-negative bacterium, and a common causative agent of community-acquired and nosocomial infections. Immunosuppressed patients, especially those in intensive care units (ICUs), are susceptible to this pathogen. Patients infected with this pathogen could be induced to develop numerous pathological characteristics, including pneumonia, bacteremia, endocarditis, meningitis, and cellulitis (1). Among the numerous capsular (K) types, K1 and K2 are often associated with highly virulent strains (2, 3), and these strains are also known as hypervirulent K. pneumoniae (hvKP). To the best of our knowledge, the isolates belonging to K types K1 and K2 alone could compose over 70% to 80% of isolates from liver abscesses (4), and they constitute almost all the isolates from meningitis or complications of endophthalmitis (5–7). Unlike classic K. pneumoniae (cKP), hvKP can develop metastatic spread and multisite infection (8). More threateningly, K47 and K64 isolates are frequently associated with resistance to carbapenem (9), which poses great challenges to antimicrobial therapy (9–11); such isolates are known as hypervirulent carbapenem-resistant K. pneumoniae (hv-CRKP). Thus, a broad-spectrum therapeutic agent needs to be designed urgently and developed against K. pneumoniae isolates of serotypes K1, K2, K47, and K64.

Vaccine treatment may be a better choice than antibiotic therapy, especially in the face of hv-CRKP isolates. Although traditional whole-cell K. pneumoniae vaccines have been considered a promising method to prevent respiratory and urinary infections by K. pneumoniae, the potential toxicity and limited protections among serotypes limit their widespread use (12). Other than that, capsular polysaccharides (CPS) and lipopolysaccharides (LPS) of K. pneumoniae with higher immunogenicity and better surface exposure make them attractive vaccine components; however, various capsular serotypes among K. pneumoniae (13–15) and potential high LPS toxicity limit the development of vaccines based on CPS and LPS.

Subunit vaccine based on certain proteins and with more flexible formulations is considered an ideal therapeutic strategy and has the potential to provide protection across different serotypes. However, the effectiveness of this type of vaccine exclusively depends on the selection of antigen proteins. Traditional antigen selection using empirical screening methods or immunoproteomics is laborious and expensive (16, 17). A rapid, more rational, comprehensive, and high-throughput approach is urgently needed.

Reverse vaccinology (RV) is an *in silico* method that uses only bacterial genome sequence, without any culture or empirical screening to identify protective antigens. This technology has been applied to conquering many pathogens (18–22). However, RV analysis based on the genome of a single strain or the DNA sequence of a plasmid hinders the development of vaccines that provide cross-serotype protection.

In this study, using 274 complete genomes of K. pneumoniae (containing the K serotypes KL1, KL2, KL47, and KL64), a method combining pangenome analysis and RV analysis (so-called pan-RV analysis), was used to screen the protective antigens from the core genome. Then, to conserve the immune resources of the body and make the immune response intensive, the epitopes of T and B cells were extracted from the conserved antigens to construct the multiepitope subunit vaccine. Subsequently, a series of immunological properties of the designed vaccine were evaluated, including allergenicity, antigenicity, various physicochemical properties, and immune simulation. The interactions between the structure of the contrived vaccine and two Toll-like receptors (TLRs) were elucidated by molecular docking. Also, molecular dynamics simulation was conducted to assess the stability of this multiepitope subunit K. pneumoniae vaccine in a humoral environment. Finally, codon optimization and cloning *in silico* were performed to ensure the efficiency of expression of the constructed vaccine in an Escherichia coli expression system. Overall, the results demonstrate the efficacy and reliability of the multiepitope subunit vaccine construct, which holds a strong rationale for further wet-lab validation for vaccine development tackling infections caused by the four threatening serotypes.

## RESULTS

### Pangenome analysis of complete genomes of Klebsiella pneumoniae.

**FIG 1:**
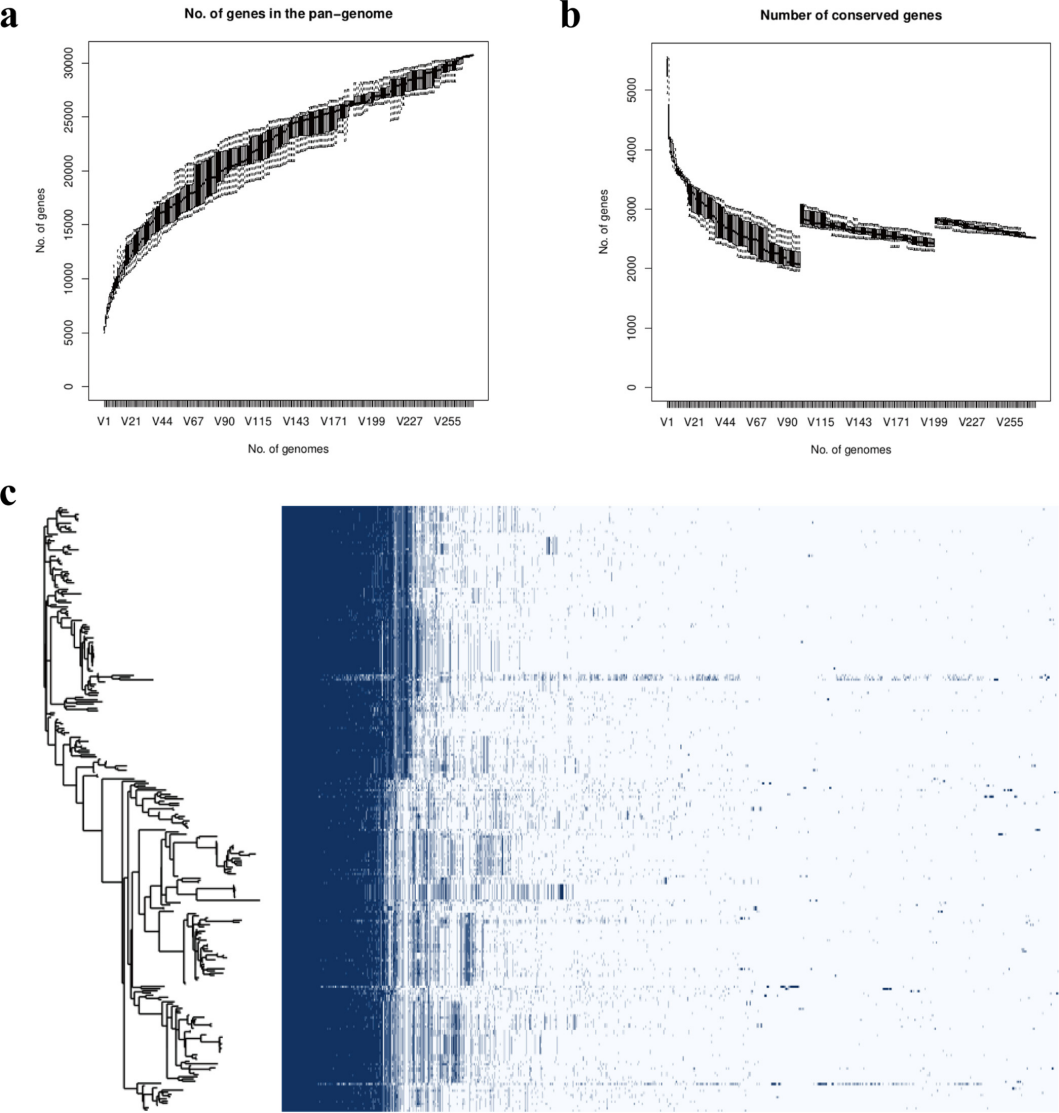
Pangenome analysis of 274 K. pneumoniae isolates. (a) The number of genes in the pangenome of K. pneumoniae increases with the number of isolates. (b) The number of conserved genes is reduced to stabilize as the number of isolates increases. (c) Whole-genome phylogenetic tree and a matrix with gene presence and absence.

Utilizing the software Prokka, a total of 274 complete genomes of K. pneumoniae were annotated, the FASTA format files of all the genomes were transformed to GFF3 format files, and these files were used to perform pangenome analysis using the program Roary. The results of pangenome analysis revealed that a total of 30,742 genes were identified among these 274 genomes; 2,519 genes were identified as the core genes. A whole-genome phylogenetic tree and a matrix with the presence and absence of core and accessory genes are shown in Fig. 1c. The pangenome of K. pneumoniae was open (shown in Fig. 1a), which means that as the number of strains analyzed increases, the total number of genes in the pangenomic pool could increase with no limit. And on the contrary, the number of conserved genes decreased and tended to be stable (shown in Fig. 1b).

### RV analysis for protein prioritization.

The core proteomes identified from the pangenome analysis were subjected to reverse vaccinology (RV) analysis to prioritize the proteins, which would be used for epitope extraction in the downstream pipeline.

### (i) Prediction of subcellular localization.

**FIG 2:**
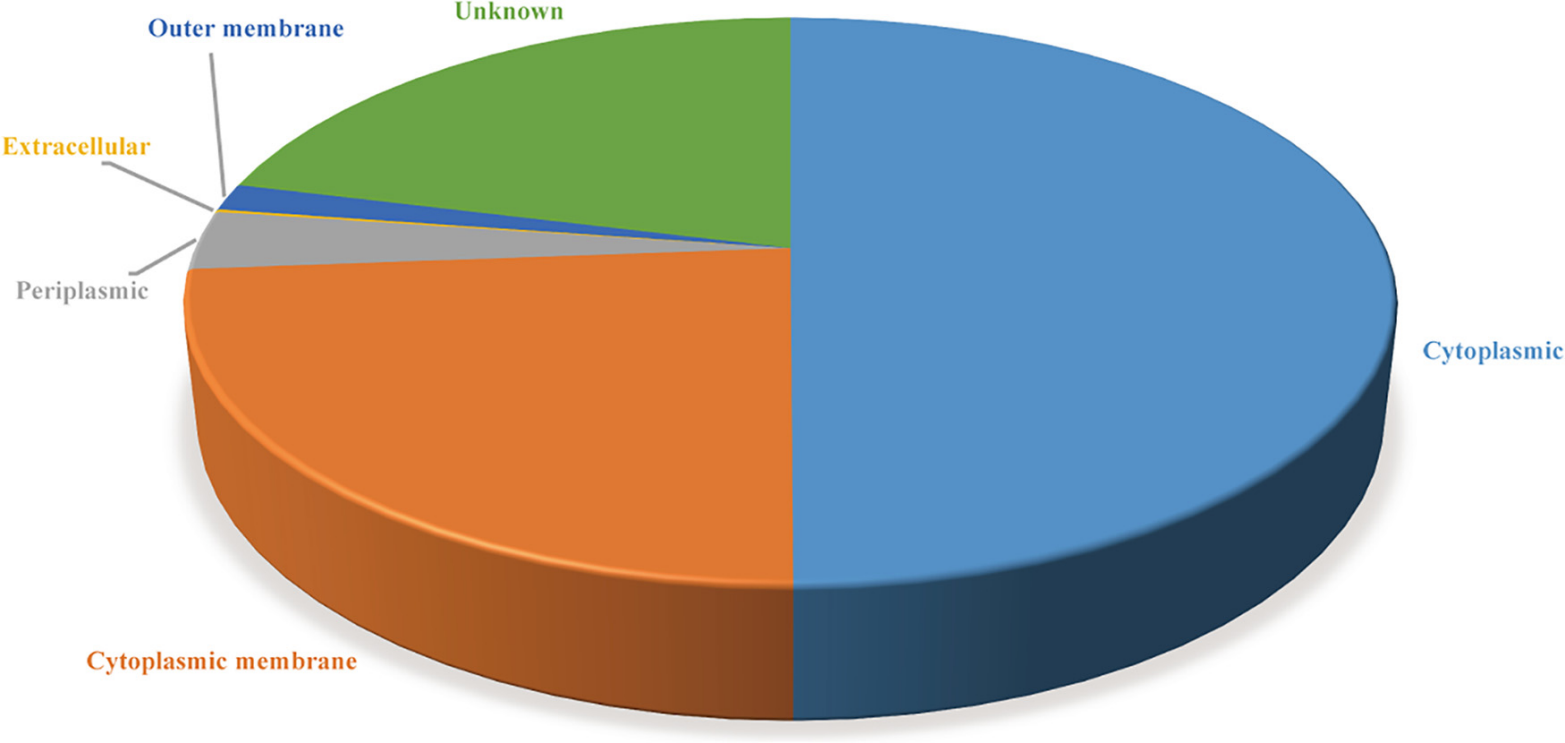
Location of proteins extracted form the core genome predicted using PSORTb.

Subcellular localization screening of the core proteomes using PSORTb (version 3.0) showed that 1,258 proteins were cytoplasmic, 601 proteins were cytoplasmic membrane, 85 were periplasmic, 4 were extracellular, 39 were outer membrane, and 532 were of unknown localization (Fig. 2). The proteins with extracellular secretion and located at the outer membrane were selected for next analysis.

### (ii) Antigenicity prediction.

The proteins identified in the upstream region were scored by VaxiJen (version 2.0). A total of 40 proteins (see Table S3 in the supplemental material) were identified as the potential antigens (with the default threshold of 0.4), of which 12 proteins (Table 1) with a score of >0.7 were screened out. These proteins were considered the prioritized proteins and were used for epitope extraction to construct the multiepitope K. pneumoniae vaccine. In addition, the serotype coverage of these 12 proteins in isolates belonging to serotypes other than the four serotypes studied is listed in Table S4.

**TABLE 1:** Description of 12 potential protective antigens with VaxiJen score of >0.7

RefSeq ID^*a*^	UniProt ID of RefSeq	Protein	Gene	VaxiJen score	No. of amino acids	Subcellular location	Annotation
WP_004144576.1	A0A0H3GIV3	PHOE	*phoE*	0.7708	350	Outer membrane	Outer membrane pore protein E
WP_002895068.1	A0A0H3GQC1	PAL	*pal_2*	0.9239	174	Outer membrane	Peptidoglycan-associated protein
Not available	J2LUK0	FEPA	*fepA*	0.7065	748	Outer membrane	Outer membrane receptor FepA
WP_002901634.1	A0A377ZI05	OMPW	*ompW*	0.7549	212	Outer membrane	Outer membrane protein W
WP_004217144.1	J2X9A3	FIU	*fiu*	0.7079	772	Outer membrane	Catecholate siderophore receptor Fiu
WP_002907749.1	A0A663AU05	SLYB	*slyB*	0.983	155	Outer membrane	Outer membrane lipoprotein SlyB
WP_002908860.1	A0A0H3GU43	LPP	*lpp*	0.7585	78	Outer membrane	Major outer membrane lipoprotein Lpp
WP_002911596.1	W1DR23	OMPN	*ompN_1*	0.7072	381	Outer membrane	Outer membrane protein N
WP_004149542.1	J2DJ81	NLPD	*nlpD_2*	0.7571	376	Outer membrane	Lipoprotein NlpD
WP_002916050.1	W1DS11	KDGM	*kdgM*	0.8903	231	Outer membrane	Oligogalacturonate-specific porin protein KdgM
WP_015959089.1	A6TF12	DAMX	*damX*	0.8546	428	Outer membrane	Cell division protein DamX
WP_002921917.1	A0A663BKZ3	YIAD	*yiaD*	0.8886	220	Outer membrane	Putative lipoprotein YiaD

a ID, identifier.

### HTL epitope prediction.

The helper T-lymphocyte (HTL) epitopes were identified using the IEDB server for three HLA supertypes, including HLA-DR (HLA-DRB1*01:01, HLA-DRB1*03:01, HLA-DRB1*04:01, HLA-DRB1*04:05, HLA-DRB1*07:01, HLA-DRB1*08:02, HLA-DRB1*09:01, HLA-DRB1*11:01, HLA-DRB1*12:01, HLA-DRB1*13:02, HLA-DRB1*15:01, HLA-DRB3*01:01, HLA-DRB3*02:02, HLA-DRB4*01:01,and HLA-DRB5*01:01), HLA-DQ (HLA-DQA1*05:01/DQB1*02:01, HLA-DQA1*05:01/DQB1*03:01, HLA-DQA1*03:01/DQB1*03:02, HLA-DQA1*04:01/DQB1*04:02, HLA-DQA1*01:01/DQB1*05:01, and HLA-DQA1*01:02/DQB1*06:02), and HLA-DP (HLA-DPA1*02:01/DPB1*01:01, HLA-DPA1*01:03/DPB1*02:01, HLA-DPA1*01:03/DPB1*04:01, HLA-DPA1*03:01/DPB1*04:02, HLA-DPA1*02:01/DPB1*05:01, and HLA-DPA1*02:01/DPB1*14:01). All the predicted T-cell epitopes are listed in Table S5. The epitopes with the lowest percentile rank (only the epitopes with percentile rank of <1 were sorted) and a 50% inhibitory concentration (IC_50_) value of <50 nM in each HLA supertype of each prioritized protein were considered HTL epitopes in our analysis pipeline. A total of 20 epitopes were screened out for our multiepitope vaccine construction (listed in Table 2).

**TABLE 2:** Predicted T-cell epitopes extracted from conserved potential antigens for the construction of multiepitope subunit vaccine

Protein	Epitope	Allele	Percentile rank	IC_50_ (nM)
PHOE	MMGFVASTATQAAEV	HLA-DRB1*09:01	0.11	8.9
PAL_2	QNNIVYFDLDKYDIR	HLA-DQA1*01:01/DQB1*05:01	0.62	47.1
	DKYDIRSDFAAMLDA	HLA-DRB3*01:01	0.05	18
FEPA	FNVPFFWLADQTLTL	HLA-DQA1*05:01/DQB1*02:01	0.73	39.2
	IPGIRFDYHNQFGSN	HLA-DRB3*01:01	0.05	20.6
OMPW	GINYTTFFNEDFNDT	HLA-DPA1*01:03/DPB1*02:01	0.85	30.5
	VRPYVGAGINYTTFF	HLA-DQA1*05:01/DQB1*03:01	0.19	13.4
	RLDPWVFMFSAGYRF	HLA-DRB1*07:01	0.27	5.8
FIU	LCLGASPAAGIAAEN	HLA-DQA1*05:01/DQB1*03:01	0.39	18.3
	GGGVRYVGSLRRGSD	HLA-DRB5*01:01	0.37	3.3
LPP1	None			
SLYB	SNAIGAIGGAVLGGF	HLA-DQA1*05:01/DQB1*03:01	0.15	18.3
	SVTYGTIVHTRAVQI	HLA-DRB1*07:01	0.07	2.9
OMPN_1	KRKVLALMVPALLMA	HLA-DRB1*01:01	0.16	34.2
NLPD_2	APVSSAGGAASSSTN	HLA-DQA1*05:01/DQB1*03:01	0.45	19.2
	AQPIQPMQTQTIQPA	HLA-DRB4*01:01	0.08	33
KDGM	HLHAQYSFDNGFYVA	HLA-DRB3*01:01	0.12	5.7
DAMX	PAATAAAAAPAAKTG	HLA-DQA1*05:01/DQB1*03:01	0.02	6
	LDKYVVYETSRNGQP	HLA-DRB1*04:05	0.65	30.9
YIAD	GKGALIGAAAGAALG	HLA-DQA1*05:01/DQB1*03:01	0.02	13.9
	GDNIVLNMPNNVTFD	HLA-DRB1*13:02	0.01	1.3

### B-cell epitope prediction.

The ABCpred and BCPred servers were used to identify the B-cell epitopes. The predicted epitopes were compared, and the overlaps between the predictions of the two servers were used for multiepitope vaccine construction. The epitope identification threshold of ABCpred is set to 0.5, and epitopes screened out by the BCPred server with classifier specificity set to 75%. All the predicted B-cell epitopes are listed in Table S6. The epitope (16-mer) that overlapped between the two servers with the highest score (based on the score obtained by the ABCpred server) in each protein was selected (Table 3) for vaccine construction.

**TABLE 3:** Predicted B-cell epitopes extracted from conserved potential antigens for the construction of multiepitope subunit vaccine

Protein	Epitope	Location in the protein (residue)	ABCpred score
PHOE	SEFSGNKTESDSSQKT	79	0.87
PAL_2	PVMAIAACSSNKNASN	15	0.93
FEPA	QGNIYAGDTQYSNGNL	273	0.92
OMPW	TATVRPTEGSDNVLGS	33	0.9
FIU	RYHPGEPRTFMLTANV	744	0.94
SLYB	IGAIGGAVLGGFLGNT	62	0.89
LPP1	None		
OMPN_1	AGSGEGTNNGGKRKLA	179	0.87
NLPD_2	SGMLITPPPSGVKSAP	49	0.94
KDGM	TVEAKWRSGGDNGSQP	56	0.92
DAMX	PQAVAKTPVESKPVQP	257	0.91
YIAD	RTTGMGPANPIASNST	187	0.86

### Multiepitope subunit vaccine design.

The HTL epitopes and B-cell epitopes analyzed upstream were fused with linker sequences. The HTL epitopes were linked by a GPGPG linker, the B-cell epitopes were linked by a KK linker, and the cholera toxin subunit B (CTB) was employed as an adjuvant which was linked to the N terminus of the construct with the help of an EAAAK linker. The amino acid sequence of our developed multiepitope K. pneumoniae vaccine is as follows: MMGFVASTATQAAEVGPGPGQNNIVYFDLDKYDIRGPGPGDKYDIRSDFAAMLDAGPGPGFNVPFFWLADQTLTLGPGPGIPGIRFDYHNQFGSNGPGPGGINYTTFFNEDFNDTGPGPGVRPYVGAGINYTTFFGPGPGRLDPWVFMFSAGYRFGPGPGLCLGASPAAGIAAENGPGPGGGGVRYVGSLRRGSDGPGPGSNAIGAIGGAVLGGFGPGPGSVTYGTIVHTRAVQIGPGPGKRKVLALMVPALLMAGPGPGAPVSSAGGAASSSTNGPGPGAQPIQPMQTQTIQPAGPGPGHLHAQYSFDNGFYVAGPGPGPAATAAAAAPAAKTGGPGPGLDKYVVYETSRNGQPGPGPGGKGALIGAAAGAALGGPGPGGDNIVLNMPNNVTFDGPGPGSEFSGNKTESDSSQKTKKIGAIGGAVLGGFLGNTKKPVMAIAACSSNKNASNKKRTTGMGPANPIASNSTKKSGMLITPPPSGVKSAPKKTATVRPTEGSDNVLGSKKAGSGEGTNNGGKRKLAKKTVEAKWRSGGDNGSQPKKPQAVAKTPVESKPVQPKKRYHPGEPRTFMLTANVKKQGNIYAGDTQYSNGNLEAAAKMIKLKFGVFFTVLLSSAYAHGTPQNITDLCAEYHNTQIYTLNDKIFSYTESLAGKREMAIITFKNGAIFQVEVPGSQHIDSQKKAIERMKDTLRIAYLTEAKVEKLCVWNNKTPHAIAAISMAN.

### Prediction of allergenicity, antigenicity, and various physicochemical properties.

The allergenicity of the vaccine was evaluated using the AllergenFP (version 1.0) and AllerTOP (version 2.0) servers; the results of both servers indicated that the sequence of vaccine corresponds to a probable nonallergen. The antigenicity of the vaccine was revalidated using the VaxiJen server, and the score of antigenicity obtained by the server was 0.9223 (a protein with a score above 0.4 is predicted as a probable antigen).

Various physicochemical properties were assessed utilizing the web server ProtPara; the results of assessment are as follows. Our designed vaccine consists of 725 amino acids with 44 negatively charged residues and 69 positively charged residues, and its molecular weight is 73.9 kDa. The theoretical pI of vaccine protein is predicted to be 9.62. The vaccine contains 10,322 atoms, and its chemical formula is written as C_3277_H_5117_N_921_O_987_S_20_. The estimated half-life is 30 h *in vitro*, and it could reach more than 20 h and 10 h in yeast (*in vivo*) and in Escherichia coli (*in vivo*), respectively. The instability index (II) was computed to be 30.53, classifying the vaccine as a stable protein. The aliphatic index was calculated to be 62.68, suggesting its relatively higher thermostability. The grand average of hydropathicity (GRAVY) value of the vaccine is −0.336, indicating the hydrophilicity of the protein.

### Immune simulations *in silico*.

The C-ImmSim server was utilized for performing immune simulation and generating the immune response profile of the designed vaccine. Three injections were conducted at 4-week intervals, and a total of 200 days of immunological data were recorded.

**FIG 3:**
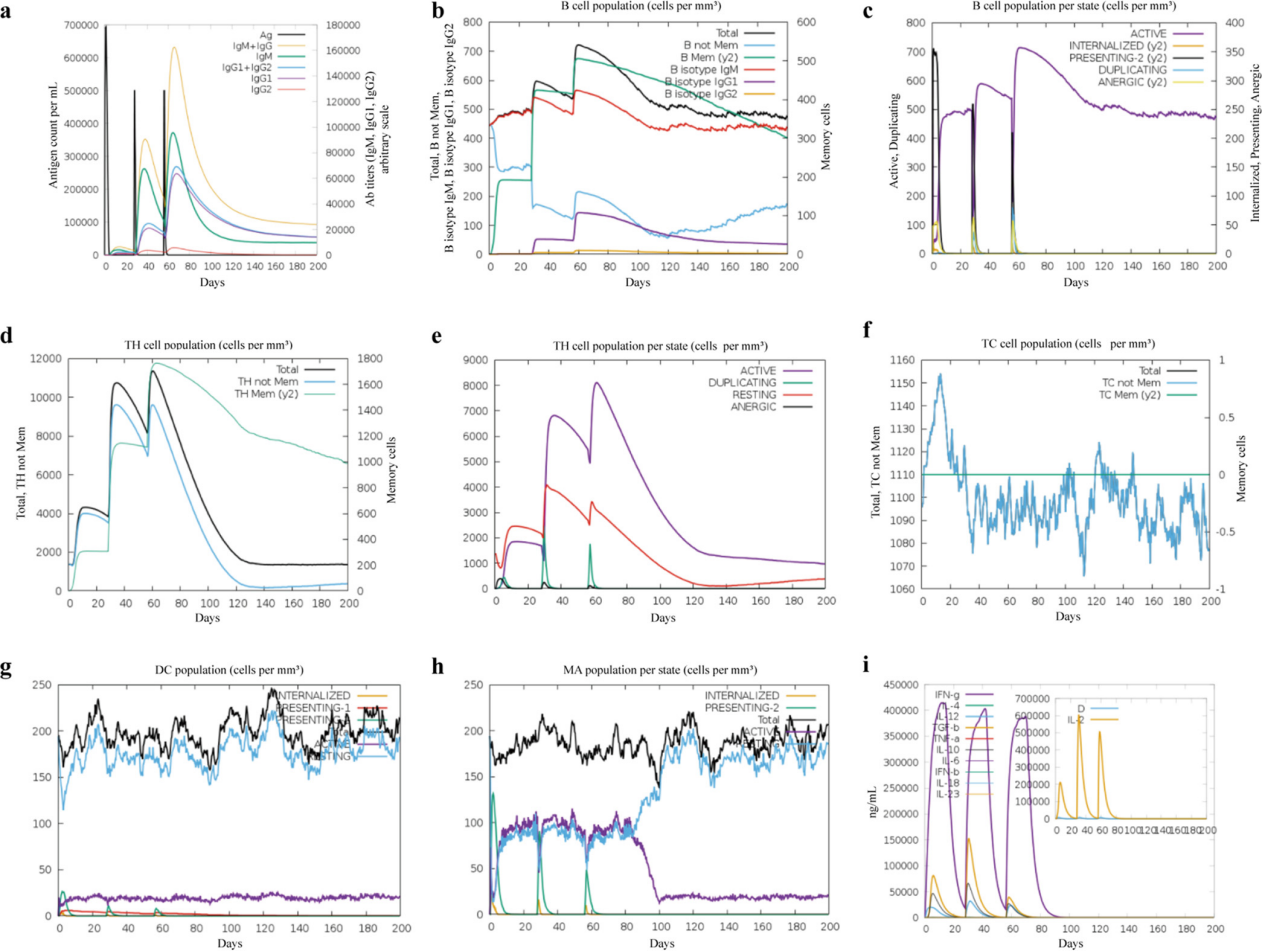
Immune simulation *in silico*. (a) Antigen and antibody levels in response to vaccine injection. (b) B-lymphocyte population after three injections of the vaccine. (c) B-lymphocyte population at each stage. (d) T-lymphocyte population after two injections of the vaccine. (e) T-lymphocyte population at each stage. (f) The population of CD8 T-cytotoxic lymphocytes at each state. (g) The population of dendritic cells at each state. The bars in the legend indicate “INTERNALIZED,” “PRESENTING-1,” “PRESENTING-2,” “Total,” “ACTIVE,” and “RESTING,” respectively. (h) Macrophage population at each state. The bars in the legend indicate “INTERNALIZED,” “PRESENTING-2,” “Total,” “ACTIVE,” and “RESTING,” respectively. (i) Concentration of cytokines and interleukins, “D” in the inset plot indicates the danger signal.

The simulation analysis showed that the antibody (Ab) titer had two steep rises after two later administrations (Fig. 3a), indicating that a strong humoral immune response was generated. Compared with the primary immune response, the secondary immune response was largely augmented, with more active B cells (Fig. 3b and c), helper T cells (Fig. 3d and e), and cytotoxic T cells (Fig. 3f). Furthermore, the increased concentration of dendritic cells (Fig. 3g) and macrophages (Fig. 3h) indicated a good antigen representation by these antigen-presenting cells (APCs). Importantly, the concentration of cytokines and interleukins is recorded in Fig. 3i, showing the relatively higher levels after the second injection. Overall, our designed vaccine has the potential ability to induce high levels of Ab, activated B cells and T cells, cytokines, and APCs against the pathogen.

### Prediction, refinement, and quality assessment of the 3D structure of the developed multiepitope subunit vaccine.

**FIG 4:**
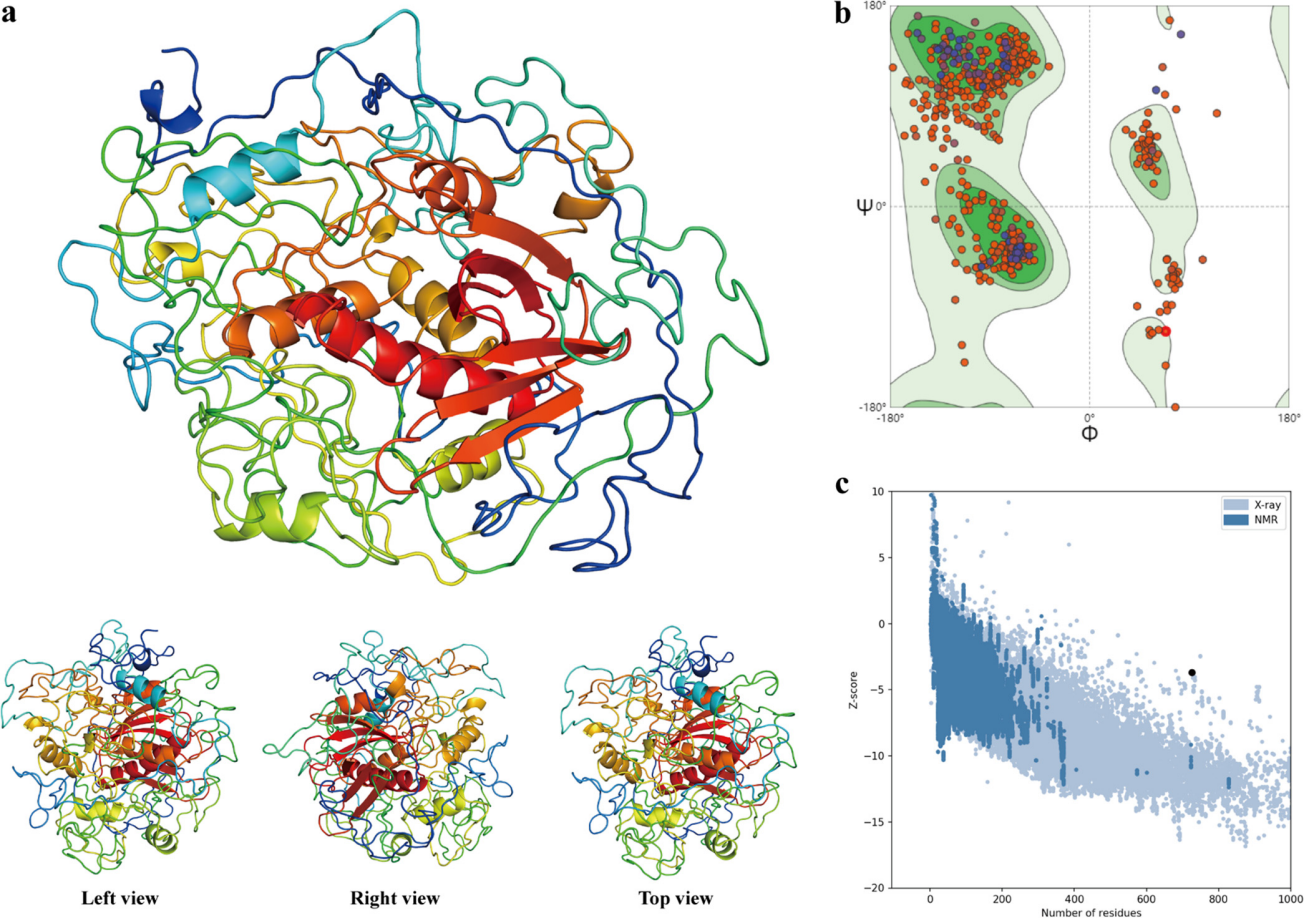
Three-dimensional structure prediction and quality assessment. (a) The 3D structure of the developed vaccine predicted using the Phyre 2 server. (b) Ramachandran plot of the final structure of the developed vaccine. (c) Z-score plot for the 3D structure of the final vaccine.

The three-dimensional (3D) structure of the designed vaccine was constructed using the Phyre 2 server, and subsequently, the initial structure was refined by the Galaxyrefine server. A model with the highest Rama favored score (80.5) was adopted among the five generated models as the final structure for downstream analysis, and the structure of this model was visualized by PyMOL (Fig. 4a).

A model with an ideal state should have more residues in the Ramachandran-favored region and have fewer in the outlier region and rotamer region; the Ramachandran plot of the final structure of our developed vaccine revealed that there were 80.50% of residues in the Ramachandran-favored region, only 3.73% in the outlier region, and 0.95% in the rotamer region (Fig. 4b). The MolProbity and Clash scores were 2.12 and 7.06, respectively. Furthermore, the overall quality and potential error of the final structure were evaluated by ProSA, and the Z-score was calculated to be −3.7, the point indicating that the vaccine protein fell with the range of experimental structures (Fig. 4c).

### Molecular dynamics simulation of the multiepitope subunit vaccine.

**FIG 5:**
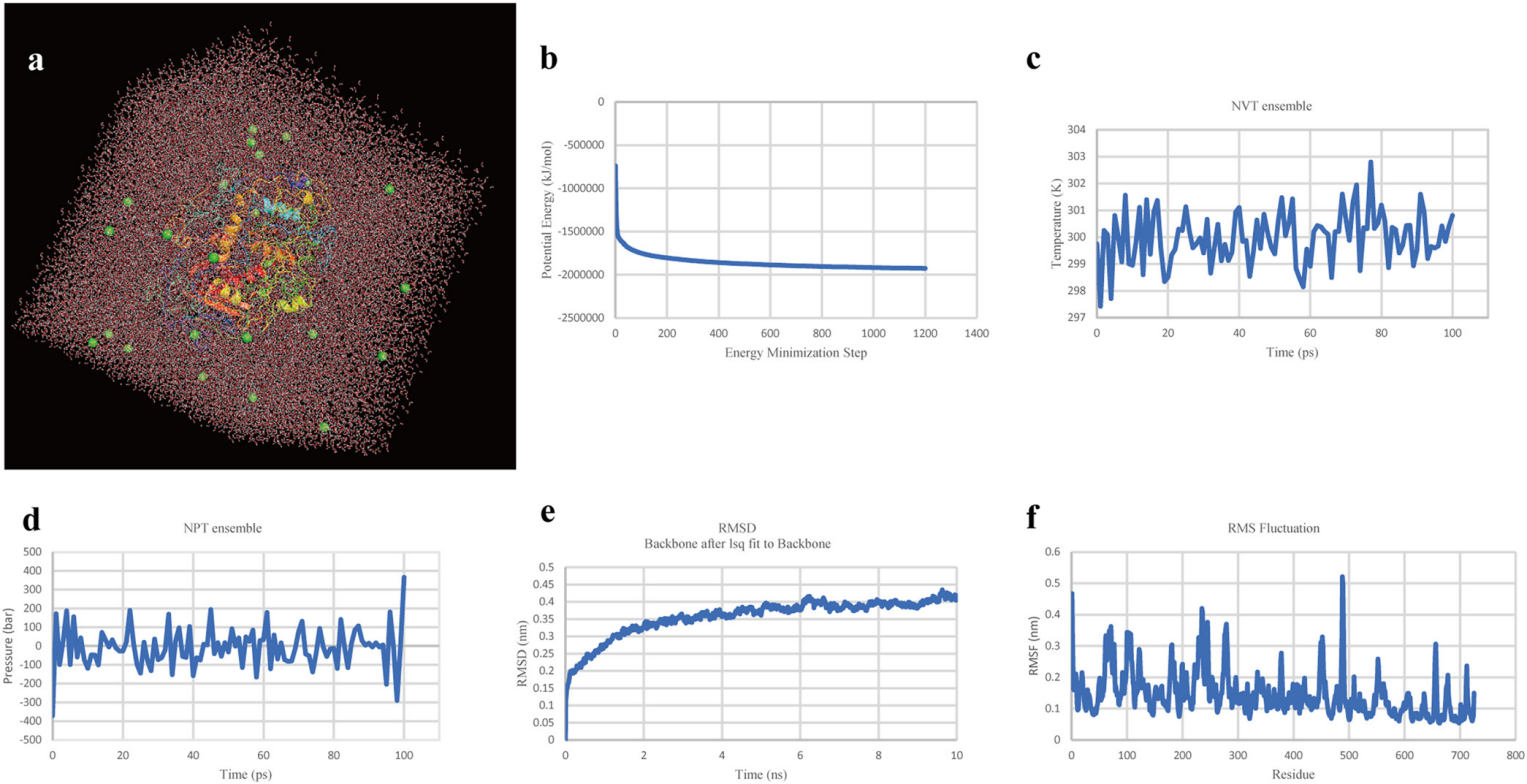
Molecular dynamics simulation. (a) The final vaccine protein in the cubic water box for molecular dynamics simulation; green balls represent sodium ions. (b) The process of system energy minimization. (c) NVT ensemble. (d) NPT ensemble. (e) Root mean square deviation (RMSD) plot reflect the stability of the final vaccine. (f) Root mean square fluctuation (RMSF) reflecting the flexibility and fluctuation of the certain regions of the final vaccine.

**FIG 6:**
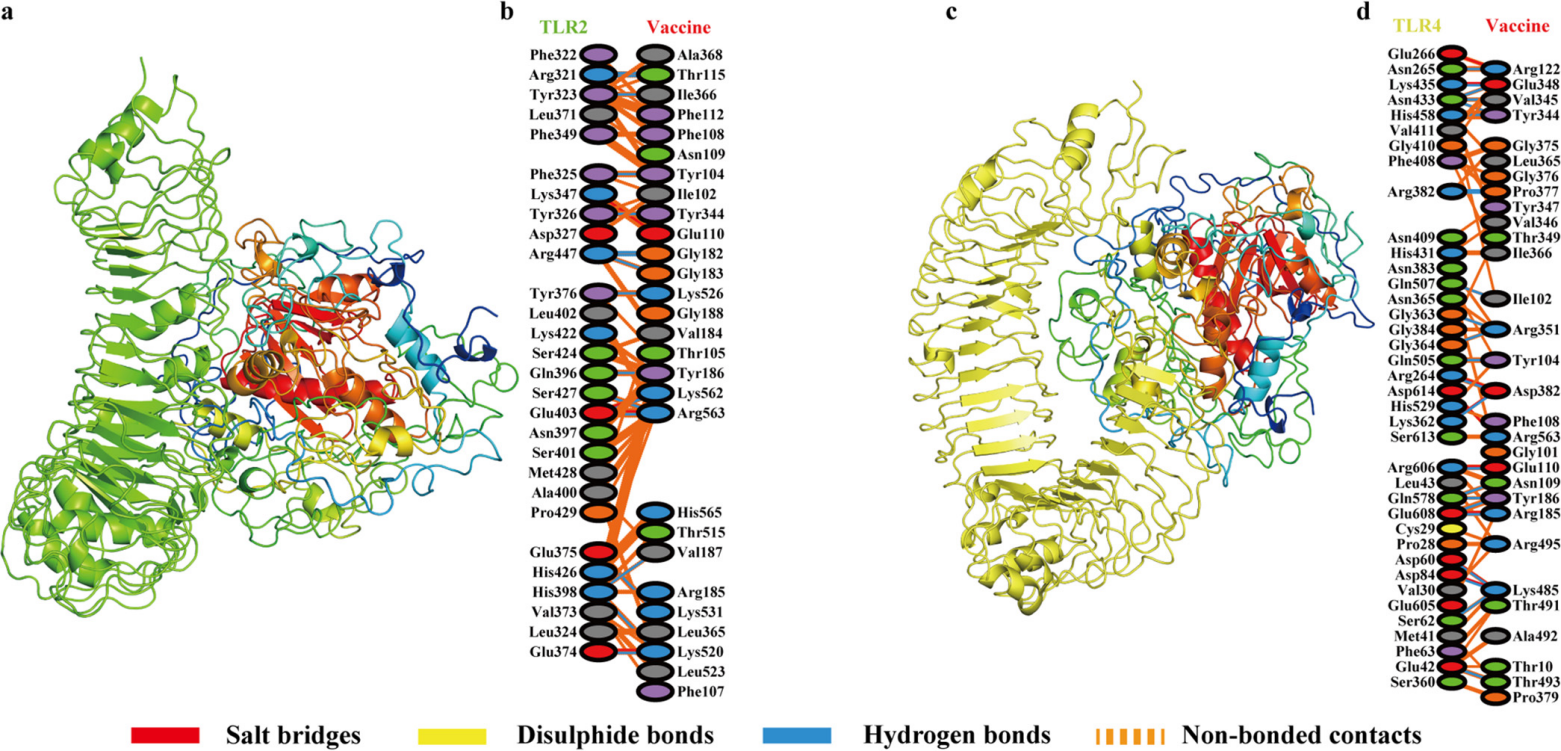
Molecular docking of designed vaccine with Toll-like receptors. (a) Vaccine-TLR2 docked complex. (b) Residue interaction between vaccine and TLR2. (c) Vaccine-TLR4 docked complex. (d) Residue interaction between vaccine and TLR4.

The molecular dynamics of the final vaccine protein was simulated using GROMACS to predict its stability in the biological environment. The OPLS-AA (Optimized Potential for Liquid Simulation - All Atom) force field was applied, and the vaccine protein was placed in the center of the cubic box with a total of 34,020 water molecules added (Fig. 5a). To ensure that the system was electrically neutral, 25 chloride ions were added into the box (Fig. 5a). Subsequently, energy minimization was performed toward the solvated and electroneutral system, using the steepest-descent minimization algorithm; the system energy was eventually reduced to around −2,000,000 KJ mol^−1^ (Fig. 5b). After that, NVT ensemble (Fig. 5c) and NPT ensemble (Fig. 5d) were conducted with settings of 300K and 1 atm, respectively. The root mean square deviation (RMSD) of the vaccine showed that the RMSD value was kept around 0.4 nm after 6 ns (Fig. 5e), indicating that the conformation of our designed vaccine was stable. The results of root medium square fluctuation (RMSF) (Fig. 6f) indicated that residues 300 to 400 and 500 to 725 have relatively lower structure flexibility, with a lower RMSF value, while the region of residues 400 to 500 has higher flexibility, with a higher RMSF value.

### Molecular docking of the multiepitope subunit vaccine structure with Toll-like receptors.

The ClusPro server was utilized for performing molecular docking of the designed vaccine with TLR2 and TLR4, respectively. For each docking, a total of 30 clusters were generated by the server; the cluster with the lowest energy score was considered the docking result. The lowest energy score of the vaccine-TLR2 docking cluster was −1,315.4, while that of the vaccine-TLR4 docking cluster was −1,264.1. The results of molecular docking of vaccine with TLR2 and TLR4 were visualized by PyMOL (Fig. 6a and c).

To further understand the interaction between vaccine and the TLRs, the PDBsum tool was used to reveal the residues and binding forces of receptor-ligand interactions. In the docking of vaccine and TLR2 (Fig. 6b), the number of interface residues of vaccine and TLR was 28; the interface areas of vaccine and TLR were 1,270 Å2 and 1,329 Å2, respectively. As for binding force, 5 salt bridges, 17 hydrogen bonds, and 166 nonbonded contacts were detected. In the docking of vaccine and TLR4 (Fig. 6d), the number of interface residues of the vaccine was 38, and that of TLR4 was 30. The interface areas of vaccine and TLR were 1,606 Å2 and 1,724 Å2, respectively. A total of 9 salt bridges, 23 hydrogen bonds, and 233 nonbonded contacts were found in the vaccine-TLR4 complex. The results indicate that the designed vaccine has high affinity with these two types of TLRs.

### Codon adaption and *in silico* cloning.

**FIG 7:**
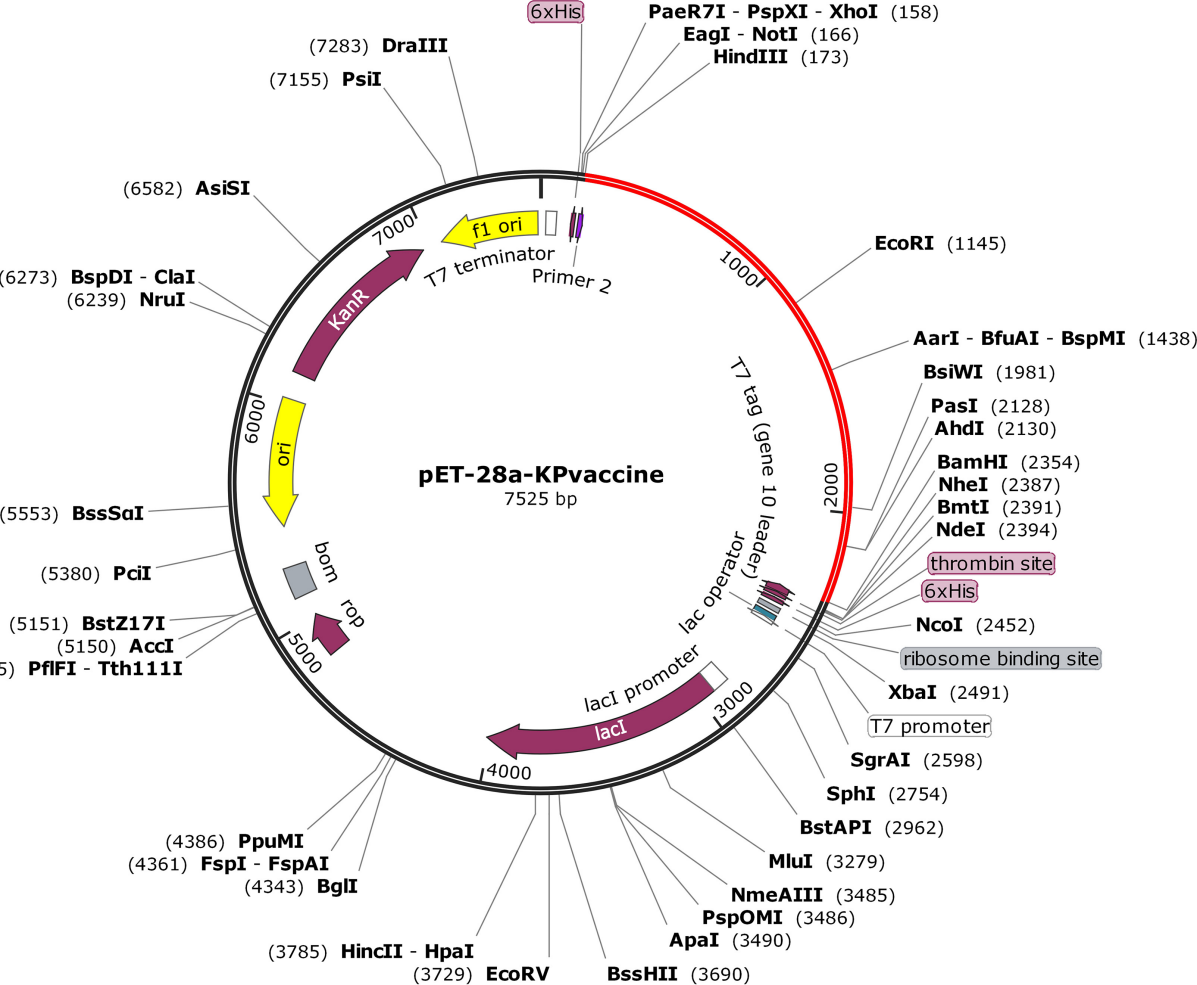
Expression vector of designed multiepitope K. pneumoniae vaccine constructed using pET28a(+). The vaccine is marked with red color, while the rest, in black, represents the pET28a(+) expression vector.

Using the JCat server, cDNA of our designed vaccine was generated and codon adaption was performed to improve the cDNA sequence (Text S7). The codon adaptation index (CAI) value and the GC content of the improved sequence were 1.0 and 54.53%, respectively, indicating high-level expression of our designed vaccine in E. coli K-12. Vector construction and visualization were performed utilizing SnapGene software; the vaccine sequence was inserted (Fig. 7, marked with red color) between the restriction sites BamHI and HindIII.

## DISCUSSION

Klebsiella pneumoniae is an opportunistic pathogen causing both community-acquired and nosocomial infections. K. pneumoniae isolates of capsular serotypes K1, K2, K47, and K64 are frequently identified as members of either of the families hvKP and hv-CRKP, which have posed significant threats to human health (2–4, 9). Vaccination is a good choice for its ability to elicit strong immune responses against drug-resistant isolates. However, conventional vaccines have many limitations; for example, the vaccines based on whole cells and LPS are limited by potential toxicity, and those of others based on CPS have poor protective ability of cross serotypes. Meanwhile, the process of traditional vaccine development is expensive, laborious, and time-consuming. Promisingly, new technologies in the field of immunoinformatics are already accelerating the development of vaccines by efficient *in silico* screening of ideal epitope candidates in a target antigen that has the potential to evoke both humoral and cellular immune responses (23). During the past few years, these technologies have also facilitated the development of epitope-based vaccines, and the immunoinformatics approach has been applied widely to design multiepitope vaccines against various pathogens (24, 25). In contrast to traditional vaccines, multiepitope vaccines have many competing advantages, such as lower reactivity, more intensive immune responses, refined stability, and improved solubility (26).

However, the majority of current multiepitope vaccines developed using immunoinformatics are based on empirical protective proteins, which limits their potential to provide broad-spectrum protection. Therefore, in this study, for the first time, an approach combining immunoinformatics and pangenome analysis was employed to develop a multiepitope subunit vaccine against K1, K2, K47, and K64 K. pneumoniae. To obtain cross-protectivity, pangenome-based reverse vaccinology (pan-RV) analysis was performed first, and a total of 12 proteins predicted as protective antigens were screened from the core genome of 274 K. pneumoniae isolates (covering the four K serotypes KL1, KL2, KL47, and KL64). Interestingly, some of these proteins have been previously confirmed as the immunological effectors among various pathogens, such as PHOE (27, 28), PAL (29, 30), NLPD (31), LPP (32), OMPW (33–35), OMPN (36, 37), and DAMX (38). More importantly, SLYB, YIAD, KDGM, FIU, and FEPA, identified in this study, may be new antigens with potential protection for cross-serotype K. pneumoniae infections. Meanwhile, more meaningfully, the results regarding the coverage of these proteins in strains other than these four serotypes suggest that they may have a more broad-spectrum protective potential. Twenty T-cell epitopes and 11 B-cell epitopes were predicted and extracted from these 12 antigen proteins, linked by the linker, and cholera toxin subunit B (CTB), with the ability to improve the antigenicity (39), was added to the N terminus to construct the vaccine.

After completing the construction of the vaccine, a series of properties were evaluated. The constructed vaccine was predicted as a nonallergen with high immunogenicity. The results of ProtParam showed that the molecular weight of the designed vaccine is 73.9 kDa, indicating that the vaccine protein is easy to express and purify. The instability index of 30.53 illustrates its stability, the aliphatic index of 62.68 indicates its higher thermostability, and the protein also has better hydrophilicity, with a GRAVY value of −0.336 (40). The immune simulations performed by C-ImmSim revealed that this constructed vaccine has the potential to elicit both humoral and cellular immune responses and induce high levels of cytokines and APCs against the pathogen. Further, the 3D structure of our developed vaccine was predicted using Phyre 2 and refined by the Galaxyrefine server. A Ramachandran plot of the final vaccine structure showed 80.5% of residues to be in the favored region, while only 3.73% were in the outlier region. The Z-score generated by ProSA is −3.7; both two indexes mentioned above indicate the high quality of the predicted 3D structure. Next, molecular dynamics simulation of this structure further proved its stability in the humoral environment, with a lower RMSD value. Additionally, considering the important role of TLR2 and TLR4 in both host-pathogen interactions (infections by K. pneumoniae could make the TLRs in human airway epithelial cells overexpressed, mainly TLR2 and TLR4 [41]) and activation of the innate immune response, molecular docking of constructed vaccine with these two Toll-like receptors was performed. The results of docking confirmed that our designed vaccine has strong interaction with TLR2 and TLR4. Finally, the cDNA of the designed vaccine was optimized for adaption for E. coli and ligated with pET-28a(+) to construct an expression vector.

Overall, the vaccine designed in this study is based on the concept of being able to provide cross-protection and was evaluated *in silico* to have ideal immunological properties. Although wet-lab validation is required to determine its safety and effectiveness, our research will pave the way for the vaccine development against these four threatening serotypes of KP isolates.

### Conclusion.

Currently, the prevalence of Klebsiella pneumoniae, especially associated with high virulence and high drug resistance, has posed significant threats to human health. In this study, a method combining pangenome analysis and immunoinformatics was employed for contriving a multiepitope subunit vaccine against the four threatening serotypes of K. pneumoniae. This designed vaccine contains multiple epitopes of T and B cells from the conserved protective antigens based on pangenome analysis. The results of immunoinformatics analysis and molecular docking indicated that it has good immunological properties and high affinity with immunoreceptors (TLR2 and TLR4). In addition, high stability in the biological environment was confirmed using molecular dynamics simulation. Finally, the cDNA of the designed vaccine was optimized and the expression vector was constructed. Despite the further wet-lab validation is required to determine its safety and effectiveness, our *in silico* research will pave the way for the vaccine development against these four threatening serotypes of K. pneumoniae.

## MATERIALS AND METHODS

**FIG 8:**
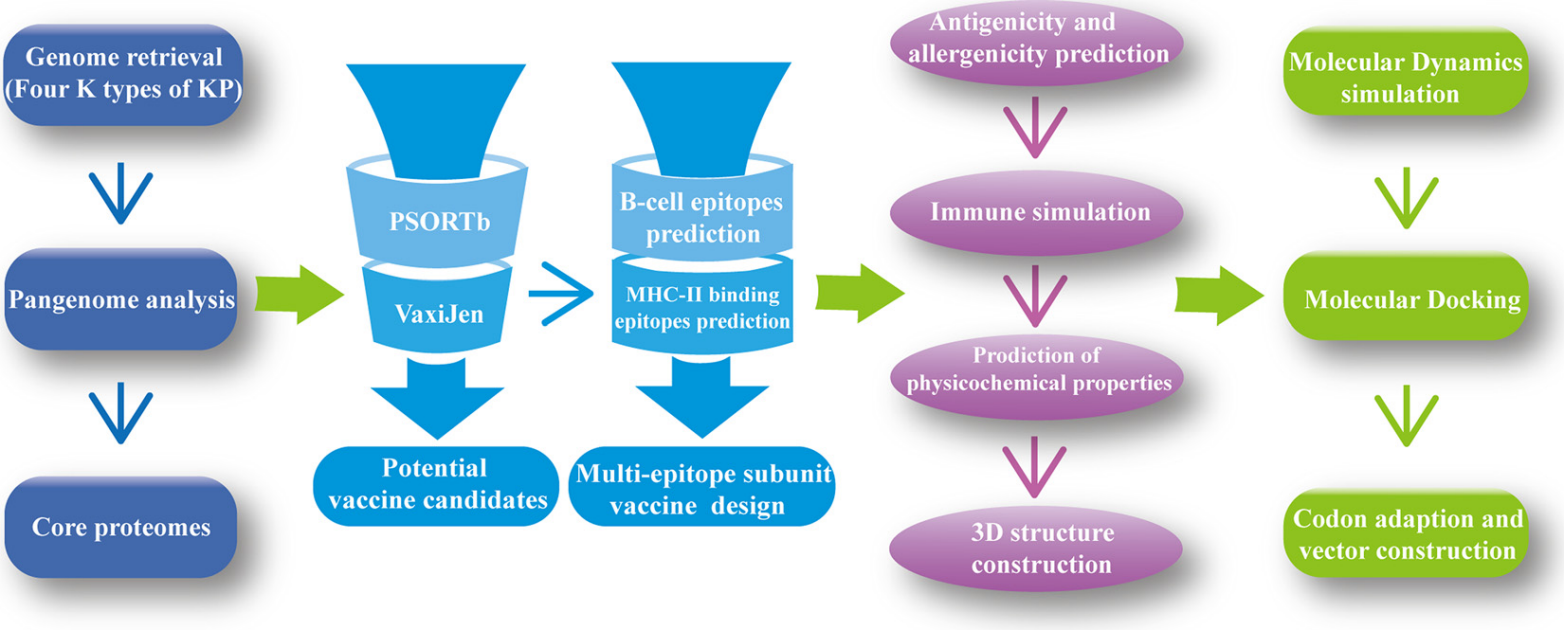
Workflow of the multiepitope KP vaccine construction based on immunoinformatics.

The workflow of our developed multiepitope KP vaccine based on immunoinformatics is illustrated in Fig. 8.

### Genome retrieval and pangenome analysis.

A total of 274 complete genomes of K. pneumoniae isolates (human isolates) encompassing K serotypes KL1, KL2, KL47, and KL64 were retrieved from the NCBI. The information for these genomes is listed in Table S1.

Using Prokka (version 1.14) (42), the coding DNA sequences (CDSs) of all the genomes were annotated, and GFF3 format files of all genome sequences were generated for pangenome analysis. Two hundred seventy-four proteomes were analyzed by Roary (version 3.13.0) (43) for identification of the core proteomes with the default settings (genes that are found in 99% to 100% of isolates were considered core genes).

### Reverse vaccinology analysis for protein prioritization.

**(i) Prediction of subcellular localization.** The core proteomes identified by pangenome analysis were uploaded to the PSORTb server (version 3.0.2; http://www.psort.org/psortb/) (44) for prediction of subcellular localization; all the settings were default and chose “Negative” in the option of “Choose Gram stain” for submission. Only the proteins predicted to be located in the outer membrane or those predicted as extracellular proteins could be screened out for the next analysis.

### (ii) Prediction of antigenicity.

The amino acid sequences of proteins filtered in previous steps were uploaded to VaxiJen (version 2.0) (http://www.ddg-pharmfac.net/vaxijen/VaxiJen/VaxiJen.html) (45) to perform antigenicity prediction. This prediction method, based on physicochemical properties of proteins, does not recourse to sequence alignment, the precision rate of which ranges from 70% to 89%. In order to maximize screening efficiency, proteins with a VaxiJen score greater than 0.7 were considered the prioritized proteins for epitope extraction.

In addition, serotype coverage of these prioritized proteins was evaluated among the K. pneumoniae isolates outside the four serotypes (accession numbers of 613 K. pneumoniae genomes are listed in Table S2).

### Helper T-lymphocyte (HTL) epitope prediction.

An online tool (MHC-II Binding Predictions) (46) in the Immune Epitope Database server (IEDB; http://www.iedb.org/) were employed for prediction of major histocompatibility complex class II (MHC-II) binding epitopes. Proteins prioritized using pan-RV strategy on core genome were uploaded to the server with binding predictions for full HLA reference set using the IEDB-recommended 2.22 prediction method. This method uses the consensus approach, combining NN-align, SMM-align, CombLib, and Sturniolo if any corresponding predictor is available for the molecule; otherwise, NetMHCIIpan is used (47–51). Prediction results were evaluated by the percentile rank and IC_50_, peptides with a small-numbered percentile rank and IC_50_ value of <50 nM were considered high affinity and used for vaccine construction.

### B-cell epitope prediction.

The ABCpred (http://crdd.osdd.net/raghava/abcpred/) and BCPRED (http://ailab.ist.psu.edu/bcpred/) servers were used for the prediction of B-cell epitopes of the prioritized proteins. The ABCpred server, based on an artificial neural network (ANN), is able to predict B-cell epitopes with 65.93% accuracy (52); default settings were applied and B-cell epitopes with scores over 0.51 were identified. The BCPred server is based on a support vector machine (SVM) algorithm using string kernels for B-cell epitope prediction (53). The same proteins as uploaded to the ABCpred server were uploaded to the BCPred server; all settings were default except for changing epitope length to 16 to make it consistent with ABCpred analysis. The overlaps (based on the results from the ABCpred server) between the predictions of the two servers were used for multiepitope vaccine construction.

### Multiepitope subunit vaccine design.

The MHC-II binding epitopes and B-cell epitopes screened in the upstream analysis were used for multiepitope subunit vaccine design. To enable effective separation of epitopes *in vivo* and to avoid the possibility of junctional epitope formation, the HTL epitopes were linked by the GPGPG linker, and B-cell epitopes were linked by the KK linker (54). Cholera toxin subunit B (CTB), retrieved from UniProt (https://www.uniprot.org/uniprot/P01556), was employed as an adjuvant to attach the N-terminal end of the vaccine using the EAAAK linker. CTB is the nontoxic portion of cholera toxin; it has affinity with the monosialotetrahexosylganglioside (GM_1_) that is widely distributed on various cell types, such as gut epithelial cells, macrophages, dendritic cells, and B cells, which enables it to be better exposed with the immune system (39).

### Prediction of allergenicity, antigenicity, and various physicochemical properties.

Proteins with allergenicity could induce a harmful immune response, and a vaccine itself should be nonallergic. Two bioinformatics tools—AllergenFP version 1.0 and AllerTOP version 2.0—were used for allergenicity prediction. AllergenFP utilizes a novel alignment-free descriptor-based fingerprint approach to identify allergens and nonallergens, while AllerTOP bases on a K-nearest neighbor algorithm with 85.3% accuracy at 5-fold cross-validation (55, 56).

The amino acid sequence of the multiepitope vaccine was uploaded to VaxiJen version 2.0 (http://www.ddg-pharmfac.net/vaxijen/VaxiJen/VaxiJen.html) to evaluate the antigenicity of the designed vaccine.

To assess the physicochemical properties of the vaccine, the web server ProtParam (https://web.expasy.org/protparam/) was employed (57), and the parameters, including the molecular weight, theoretical pI, amino acid composition, atomic composition, extinction coefficient, estimated half-life, instability index, aliphatic index, and grand average of hydropathicity (GRAVY) were computed. Molecular weight and theoretical pI are calculated from the input sequence; the amino acid and atomic compositions are self-explanatory. The extinction coefficient indicates light absorption of a protein at a certain wavelength; it is estimated by amino acid composition. *In vivo* half-life evaluation of proteins relied on the principle of “N-end rule” (58). The instability index estimates the stability of protein in a test tube; the protein is considered stable when its instability index in smaller than 40. The aliphatic index reflects the relative volume occupied by aliphatic side chains in a certain protein, which is regarded as a positive factor associated with thermostability of globular proteins. The GRAVY value for a protein is calculated as the sum of hydropathy values (59), indicating the hydrophobic nature of the protein.

### Immune simulation.

*In silico* immune simulation was performed to predict immune response profile using the C-ImmSim server (https://kraken.iac.rm.cnr.it/C-IMMSIM/) (60). C-ImmSim is an agent-based simulator of the immune response that utilizes the Celada-Seiden model to simulate the mammalian immune system against constructive vaccine to generate the immune profiles, both humoral and cellular. All the settings remain default except for setting “Simulation Steps” to 600 and the injection interval to 1 month (three injections administered).

Finally, to avoid inducing autoimmunity, the homology between designed vaccine and human protein was checked using BLASTp online server (https://blast.ncbi.nlm.nih.gov/Blast.cgi?PROGRAM=blastp&PAGE_TYPE=BlastSearch&LINK_LOC=blasthome) (61). Ideally, the designed vaccine should have no homology with human proteins.

### Prediction, refinement, and quality assessment of the 3D structure of the developed multiepitope subunit vaccine.

The Phyre 2 server (http://www.sbg.bio.ic.ac.uk/phyre2/html/page.cgi?id=index) was used to predict the three-dimensional structure of the designed vaccine; all settings on the webpage remained default except for changing the modeling mode from “normal” to “intensive.” This optional “intensive mode” combining multiple template modeling with simplified *ab initio* folding simulation could create a complete full-length model of the uploaded sequence (62).

To improve the conformational structure of the predicted protein, the 3D structure of designed vaccine modeled by Phyre 2 server was further refined using the GalaxyRefine server (http://galaxy.seoklab.org/cgi-bin/submit.cgi?type=REFINE). After performing repeated structure perturbation and global structure relaxation by molecular dynamics simulation, a total of five models of the multiepitope vaccine were generated, and among them, structure perturbation was applied only to clusters of side chains in model 1, while more aggressive perturbation to secondary-structure elements and loops were applied in models 2 to 5 (63).

The PDB file of refined structure of our developed vaccine was uploaded to the SWISS-MODEL server (https://swissmodel.expasy.org/assess) (64) for tertiary-structure assessment. A Ramachandran plot was generated in the analysis results; this plot indicates the energetically favored regions for backbone dihedral angles against of amino acid residues in protein structure. In addition, due to MolProbity-based running, the most relevant scores were provided and the residues with low quality in the developed structure were easily identified. Then the ProSA web server (https://prosa.services.came.sbg.ac.at/prosa.php) was employed for protein structure validation. ProSA calculates an overall quality score for a certain protein structure, and a score that is outside the range characteristic for native proteins may indicate that errors exist in the structure (65).

### Molecular dynamics simulation of the multiepitope subunit vaccine.

In order to further assess the stability of our developed vaccine in a biological environment, a command line-based software, GROMACS (version 2020) (66), was used. The parameter settings and analysis steps are as follows. (i) The PDB file of the designed vaccine containing only protein atoms was input into a GROMACS module called pdbgmx. The OPLS-AA (Optimized Potential for Liquid Simulation - All Atom) force field was selected and a gro file compatible with it was generated. (ii) The box type was defined as a cube and the distance from vaccine protein to box edge was set to >1.0 nm using the editconf module. Then the solvate module was used to fill the box with water. And after that, the genion tool was employed for adding ions to keep the system electrically neutral. (iii) Energy minimization was performed to ensure that the system had no steric clashes or inappropriate geometry using the steepest-descent minimization algorithm, with which the energy of the system will rapidly decrease to below 1,000 KJ mol^−1^ nm^−1^ within 5,000 maximum steps. (iv) Two phases of equilibration (NVT and NPT) were conducted to equilibrate the solvent and ions around the vaccine protein. The first phase was performed under the NVT ensemble with the restrained system heated to 300K under constant volume conditions in 100 ps. After reaching the set temperature, the second phase was imposed under the NPT ensemble using the Parrinello-Rahman pressure coupling method with a 10^5^-Pa pressure setting; the simulation steps were same as that in NVT ensemble with 100 ps. (v) Molecular dynamics simulation was carried out toward the final system for 10 ns, with coordinates recorded every 10 ps.

### Molecular docking of the multiepitope subunit vaccine structure with Toll-like receptors.

To evaluate the binding affinity between our contrived vaccine and human Toll-like receptors (TLR), TLR2 and TLR4 were separately docked with vaccine protein. The structure of TLR2 was obtained from PDB code 2Z7X, while that of TLR4 was retrieved from PDB code 3FXI. A web-based tool called ClusPro (version 2.0) (https://cluspro.bu.edu/) (67) was utilized for molecular docking; this tool uses three computational steps for protein-protein docking. First, rigid body docking is performed by sampling billions of conformations. Then the root mean square deviation (RMSD) of the 1,000 clusters with the lowest energy structures is calculated to match the most likely model of the complex. Finally, the selected model is applied with energy minimization to refine the structure, and the top 10 docking results are listed separately. The 3D structures of Toll-like receptors and the vaccine-TLR complex generated from molecular docking were visualized using PyMOL software (version 2.4.0). PDBsum (68) was used to map the amino acid residues that interacted between the contrived vaccine and TLRs.

### Codon adaption and *in silico* cloning.

A JAVA-based tool called JCat (http://www.jcat.de/Start.jsp) (69) was used for codon optimization to adapt the codon usage by the prokaryotic engineered bacteria (Escherichia coli). The amino acid sequence of the designed vaccine was pasted into the input window, the type of pasted sequence was set to “Protein sequence,” and the prokaryotic organism was chosen as Escherichia coli K-12. The vaccine-adapted DNA sequence was ligated in the multiple-cloning site (MCS) of E. coli plasmid pET-28a(+) to express the vaccine protein using SnapGene (version 4.2.4) (https://www.snapgene.com/).
