# Supplementary material for: Combing Immunoinformatics with Pangenome Analysis To Design a Multiepitope Subunit Vaccine against Klebsiella pneumoniae K1, K2, K47, and K64
Source: Microbiol Spectr. 2022 Jul 12;10(4):e01148-22. doi: 10.1128/spectrum.01148-22 (PMC9431259; doi:10.1128/spectrum.01148-22)
Supplement: Supplemental file 7 — Supplemental material. Download spectrum.01148-22-s0007.pdf, PDF file, 0.05 MB [file spectrum.01148-22-s0007.pdf]

Textfile S7 The cDNA sequence of the designed multi-epitope subunit vaccine.

>The cDNA sequence of the designed multi-epitope subunit vaccine
ATGATGGGTTTCGTTGCTTCTACCGCTACCCAGGCTGCTGAAGTTGGTCCGGGTCCGGG
TCAGAACAACATCGTTTACTTCGACCTGGACAAATACGACATCCGTGGTCCGGGTCCG
GGTGACAAATACGACATCCGTTCTGACTTCGCTGCTATGCTGGACGCTGGTCCGGGTCC
GGGTTTCAACGTTCCGTTCTTCTGGCTGGCTGACCAGACCCTGACCCTGGGTCCGGGT
CCGGGTATCCCGGGTATCCGTTTCGACTACCACAACCAGTTCGGTTCTAACGGTCCGGG
TCCGGGTGGTATCAACTACACCACCTTCTTCAACGAAGACTTCAACGACACCGGTCCG
GGTCCGGGTGTTCGTCCGTACGTTGGTGCTGGTATCAACTACACCACCTTCTTCGGTCC
GGGTCCGGGTCGTCTGGACCCGTGGGTTTTCATGTTCTCTGCTGGTTACCGTTTCGGTC
CGGGTCCGGGTCTGTGCCTGGGTGCTTCTCCGGCTGCTGGTATCGCTGCTGAAAACGG
TCCGGGTCCGGGTGGTGGTGGTGTTCGTTACGTTGGTTCTCTGCGTCGTGGTTCTGACG
GTCCGGGTCCGGGTTCTAACGCTATCGGTGCTATCGGTGGTGCTGTTCTGGGTGGTTTC
GGTCCGGGTCCGGGTTCTGTTACCTACGGTACCATCGTTCACACCCGTGCTGTTCAGAT
CGGTCCGGGTCCGGGTAAACGTAAAGTTCTGGCTCTGATGGTTCCGGCTCTGCTGATG
GCTGGTCCGGGTCCGGGTGCTCCGGTTTCTTCTGCTGGTGGTGCTGCTTCTTCTTCTAC
CAACGGTCCGGGTCCGGGTGCTCAGCCGATCCAGCCGATGCAGACCCAGACCATCCA
GCCGGCTGGTCCGGGTCCGGGTCACCTGCACGCTCAGTACTCTTTCGACAACGGTTTC
TACGTTGCTGGTCCGGGTCCGGGTCCGGCTGCTACCGCTGCTGCTGCTGCTCCGGCTG
CTAAAACCGGTGGTCCGGGTCCGGGTCTGGACAAATACGTTGTTTACGAAACCTCTCG
TAACGGTCAGCCGGGTCCGGGTCCGGGTGGTAAAGGTGCTCTGATCGGTGCTGCTGCT
GGTGCTGCTCTGGGTGGTCCGGGTCCGGGTGGTGACAACATCGTTCTGAACATGCCGA
ACAACGTTACCTTCGACGGTCCGGGTCCGGGTTCTGAATTCTCTGGTAACAAAACCGA
ATCTGACTCTTCTCAGAAAACCAAAAAAATCGGTGCTATCGGTGGTGCTGTTCTGGGT
GGTTTCCTGGGTAACACCAAAAAACCGGTTATGGCTATCGCTGCTTGCTCTTCTAACAA
AAACGCTTCTAACAAAAAACGTACCACCGGTATGGGTCCGGCTAACCCGATCGCTTCT
AACTCTACCAAAAAATCTGGTATGCTGATCACCCCGCCGCCGTCTGGTGTTAAATCTGC
TCCGAAAAAAACCGCTACCGTTCGTCCGACCGAAGGTTCTGACAACGTTCTGGGTTCT
AAAAAAGCTGGTTCTGGTGAAGGTACCAACAACGGTGGTAAACGTAAACTGGCTAAA
AAAACCGTTGAAGCTAAATGGCGTTCTGGTGGTGACAACGGTTCTCAGCCGAAAAAA
CCGCAGGCTGTTGCTAAAACCCCGGTTGAATCTAAACCGGTTCAGCCGAAAAAACGTT
ACCACCCGGGTGAACCGCGTACCTTCATGCTGACCGCTAACGTTAAAAAACAGGGTAA
CATCTACGCTGGTGACACCCAGTACTCTAACGGTAACCTGGAAGCTGCTGCTAAAATG
ATCAAACTGAAATTCGGTGTTTTCTTCACCGTTCTGCTGTCTTCTGCTTACGCTCACGG
TACCCCGCAGAACATCACCGACCTGTGCGCTGAATACCACAACACCCAGATCTACACC
CTGAACGACAAAATCTTCTCTTACACCGAATCTCTGGCTGGTAAACGTGAAATGGCTAT
CATCACCTTCAAAAACGGTGCTATCTTCCAGGTTGAAGTTCCGGGTTCTCAGCACATCG
ACTCTCAGAAAAAAGCTATCGAACGTATGAAAGACACCCTGCGTATCGCTTACCTGAC
CGAAGCTAAAGTTGAAAAACTGTGCGTTTGGAACAACAAAACCCCGCACGCTATCGC
TGCTATCTCTATGGCTAAC
